# Ultrafast acousto-optic modulation at the near-infrared spectral range by interlayer vibrations

**DOI:** 10.1515/nanoph-2023-0769

**Published:** 2024-02-02

**Authors:** Tae Gwan Park, Chaeyoon Kim, Eon-Taek Oh, Hong Ryeol Na, Seung-Hyun Chun, Sunghun Lee, Fabian Rotermund

**Affiliations:** Department of Physics, Korea Advanced Institute of Science and Technology, Daejeon 34141, Republic of Korea; Department of Physics and Astronomy, Sejong University, Seoul 02504, Republic of Korea; Division of Nanotechnology, Convergence Research Institute, Daegu Gyeongbuk Institute of Science and Technology, Daegu 42988, Republic of Korea

**Keywords:** ultrafast optics, acousto-optic modulation, photoelastic effect, coherent acoustic phonons, two-dimensional materials

## Abstract

The acousto-optic modulation over a broad near-infrared (NIR) spectrum with high speed, excellent integrability, and relatively simple scheme is crucial for the application of next-generation opto-electronic and photonic devices. This study aims to experimentally demonstrate ultrafast acousto-optic phenomena in the broad NIR spectral range of 0.77–1.1 eV (1130–1610 nm). Hundreds of GHz of light modulation are revealed in an all-optical configuration by combining ultrafast optical spectroscopy and light–sound conversion in 10–20 nm-thick bismuth selenide (Bi_2_Se_3_) van der Waals thin films. The modified optical transition energy and the line shape in the NIR band indicate phonon–photon interactions, resulting in a modulation of optical characteristics by the photoexcited interlayer vibrations in Bi_2_Se_3_. This all-optical, ultrafast acousto-optic modulation approach may open avenues for next-generation nanophotonic applications, including optical communications and processing, due to the synergistic combination of large-area capability, high photo-responsivity, and frequency tunability in the NIR spectral range.

## 1 Introduction

Light modulation in terms of amplitude, frequency, momentum, and polarization is crucial for signal processing and telecommunication in optoelectronic and photonics fields. A key method for achieving this modulation is acousto-optic (AO) modulation, which relies on the coupling of phonons and photons, causing optical constants to fluctuate due to acoustic waves in materials. Phonon–photon coupling forms the basis for commercial AO devices [1] and recent amplitude/phase modulators [2], tweezers [3], beam steering [4], nonreciprocal light propagation [5], [6], active information manipulation [7], and spatial mode conversion [8]. Conventional AO devices are typically centimeter-scale in size, employing bulk crystals and operating in the MHz range. Despite their excellent performance, current AO devices encounter challenges in compatibility with high-speed modulation in the GHz to THz range and emerging integrated on-chip systems [9], [10]. Despite achieving high-frequency modulations with integrability at a small scale, the determination of operating wavelengths requires a comprehensive study of optomechanics. For instance, investigations such as generating high-intensity phonons with the large modal overlap of acousto-optic coupling are required.

One of the most straightforward methods for this purpose is optical spectroscopy, including measurement techniques such as absorption, reflectivity, and photoluminescence during acoustic interactions [11]. However, this method lacks details regarding modulation speed, phase, and the specific phonon modes involved in the operation. An alternative approach is ultrafast optical approach, allowing the observation of phonon–photon interactions in both time and energy domains [12], [13], [14]. Ultrashort pump pulses generate acoustic waves on the material surface and inside it by increasing carrier density and lattice temperature [15]. Subsequent broadband probe pulses are then utilized to monitor the modulation of optical properties resulting from AO effects. This study on ultrafast acousto-optic modulation reveals temporal and simultaneous spectroscopic features, including operating speed, decoherence time, and modifications in optical properties through acoustic modulation of particular phonon modes.

**Figure 1:**
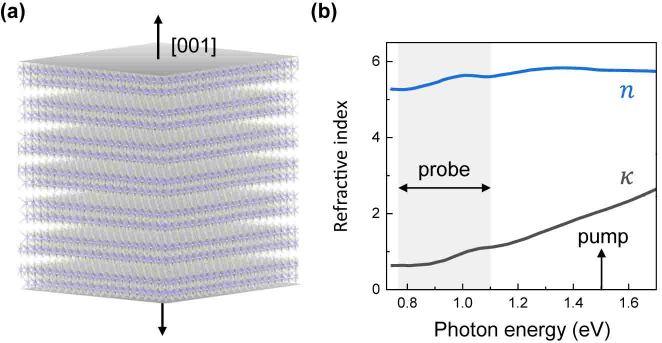
(a) Crystal structure of Bi_2_Se_3_-layered vdW thin films and interlayer modulation schematic by out-of-plane straining and (b) real (*n*) and imaginary part (*κ*) of refractive index of Bi_2_Se_3_ obtained from [16]. The pump and probe photon energies used here are indicated by the vertical arrow and the gray area, respectively.

We aim to develop a high-speed AO modulator that operates at near-infrared (NIR) wavelengths, similar to those commonly used in modern communication, capable of achieving speeds in the hundreds of GHz. We employ ultrafast optical spectroscopy with a broadband NIR probe to introduce the ultrafast light modulation via a AO coupling in bismuth selenide (Bi_2_Se_3_) van der Waals (vdW) thin films. The ultrafast pump pulses generate out-of-plane strain and interlayer vibrations as shown in Figure 1(a). A broadband NIR probe light (0.77–1.1 eV) is then applied to detect AO modulations. Our results demonstrate that changes in interlayer distance cause fluctuations in optical properties. The shift of interband transition energy at approximately 15 meV and the line shape change at 40 meV are observed through the AO effects in the NIR band. The experimental results are reproduced well by the strain-dependent density functional theory (DFT) calculations, which facilitated electronic band modulation, including energy and curvature by lattice deformation. Within the vdW gap oscillations, the modulation frequency can widely be tuned from a few GHz to sub-THz. Our findings prove the feasibility of the presented all-optical AO modulation by exploiting Bi_2_Se_3_ thin films for light modulator at NIR wavelengths with high speed and integrability in on-chip and nano-devices.

## 2 Results and discussion

In this study, high-quality Bi_2_Se_3_ vdW thin films were prepared by molecular beam epitaxy with different thicknesses on an Al_2_O_3_ (0001) substrate. The crystal structures of Bi_2_Se_3_ are rhombohedral layered structure with a unit quintuple layer (QL) (see Figure 1(a)). The QL corresponds to a thickness of 1 nm. Atomic force microscopy confirmed the number of QLs in the used sample as 13, 17, and 22 QLs. Figure 1(b) shows the real (*n*) and imaginary (*κ*) part of the refractive index of the Bi_2_Se_3_ films at the ground state, as obtained from recent spectroscopic ellipsometry for the 10-QL Bi_2_Se_3_ by Fang [16]. At the NIR range, the *κ* values became significant from 0.9 eV, indicating an absorption edge. A broad peak near 1.0 eV featured a resonant interband transition within the continuum free carriers. The peak energy near 1.0 eV was consistent with the interband transition in NIR band [16], [17].

We investigated ultrafast acousto-optic effects through transient reflectivity (TR) spectroscopy, employing a NIR pump and widely tunable NIR probe pulses. To generate these pulses, we used an 80-MHz Ti:sapphire oscillator (MAITAI, Spectra Physics) producing optical pulses with a photon energy of 1.5 eV and a pulse duration of 100 fs. This oscillator served as the pump for a synchronously pumped optical parametric oscillator (SPOPO) to generate tunable NIR probe pulses ranging from 0.77 to 1.1 eV. Approximately 60 % of the laser output power was used to pump the SPOPO, while the remaining power was used for optical pump pulses. These pump pulses excited carriers, leading to subsequent acoustic waves in van der Waals (vdW) thin films. The probe pulses were collinearly combined with the pump pulses using a dichroic mirror. The pump and probe beams were then focused on by the NIR objectives (Mitutoyo Apochromatic, 50× magnification). The pump fluence used in this work was approximately 120 μJ/cm^2^, yielding an excited carrier density (*n*_*ex*_) of 5.8 × 10^19^ cm^−3^. By comparison, the probe fluence remained to be 10^2^ times weaker than the pump fluence. The time delay was controlled using a linear translation stage. While tuning the probe wavelengths, we measured the TR (Δ*R*/*R*_0_) signal with a free-space biased Ge detector (DET50B, Thorlabs) and a lock-in amplifier (SR830, Stanford Research System), chopping the optical pulses at 290 Hz. Spectral filters that acted as long-pass filters were used to block the pump beam in the photodetector. All ultrafast spectroscopic measurements were performed at room temperature and normal pressure. We obtained the transient NIR spectra due to the wide tuning range of the probe wavelength from SPOPO.

**Figure 2:**
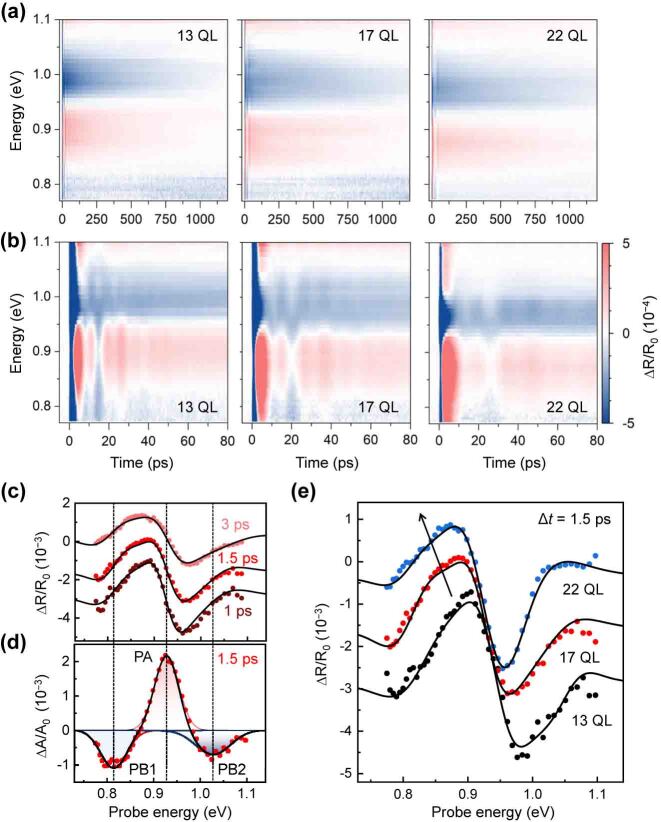
Ultrafast pump–probe spectroscopic results: (a) TR signals as a function of time and probe energy for the 13, 17, and 22 QL Bi_2_Se_3_ samples; (b) same TR signals in the (a) in short time window, which highlights spectral modulation by coherent oscillations. (c) Selected TR spectra in the 17-QL sample at 1, 1.5, and 3 ps time delay between pump and probe pulses; (d) corresponding TA spectra obtained using the Kramers–Kronig transformation; and (e) number of QLs-dependent TR spectra at 1.5 ps time delay, in which the black curves in (c–e) denote the fitting results based on the derivative-like response in the TA spectra and their Hilbert transformation result in TR spectra.

Figure 2(a) presents the TR signal for the 13-, 17-, and 22-QL Bi_2_Se_3_ films as a function of time and energy. The observed photocarrier dynamics extended over a nanosecond time frame. Figure 2(b) highlights the ultrafast response within 100 ps. Across all samples, three main features were noted: (1) initially, a negative TR was observed for all probe energies following photoexcitation. (2) Subsequently, a derivative-like differential spectrum, with a sign that changed depending on the energy due to resonant changes in optical transition, was subsequently obtained. (3) A noticeable sinusoidal modulation appeared within the incoherent background from the excited carriers.

Figure 2(c) shows the TR spectra at selected time delays. The derivative-like signal was embedded within a negative Δ*R*/*R*_0_ response across the entire energy range, persisting for 3 ps. Besides the negative TR response, the derivative-like TR feature also referred to the resonant changes in absorption derived by the Kramers–Kronig relation. We transformed the TR to the corresponding transient absorption (TA, Δ*A*/*A*_0_) spectra using an inverse Hilbert transformation (iHT) as shown in Figure 2(d). The obtained TA displayed second derivative-like spectra with a central peak of the photo-induced absorption (PA) at 0.93 eV and two satellite peaks denoted as two photobleaching (PB) peaks at 0.82 and 1.03 eV. These observed TA spectra were linked to energy shifts and broadening associated with band filling during resonant transitions. The measured peak energy of the TA spectra was consistent with the interband transition energy in the equilibrium optical spectrum [16], [17]. From the thickness-dependent TR spectra in Figure 2(e), we observed a more pronounced negative in the Δ*R*/*R*_0_ spectra in the thinner film. The characteristic spectra were redshifted according to the thickness increase. This behavior was consistent with a previous observation of the redshift in the optical transition energy as the sample thickness increased [16], [18].

**Figure 3:**
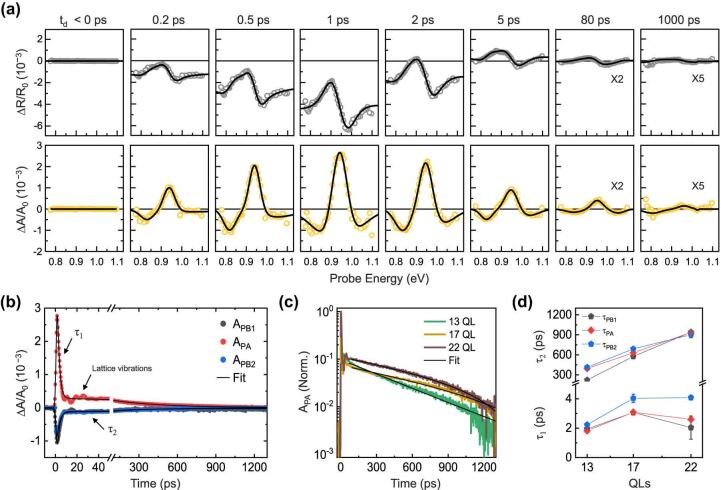
Ultrafast carrier dynamics: (a) time-dependent TR spectra and corresponding TA spectra, with black curves representing fitting results based on the Kramers–Kronig relation. (b) Pump–probe traces collected at resonant energies (PB1 and 2 and PA); (c) relaxation dynamics of excited carriers dependent on the number of QLs, with black curves indicating fitting based on the bi-exponential decay function; and (d) decay constant obtained from (c).

Figure 3(a) shows the time-dependent changes of resonant energies, obtained from the TR spectra and the corresponding TA spectra. The transient spectrum features indicated the ultrafast nonequilibrium dynamics during 5 ps and slow recovery. Temporal traces at three representative peaks showed a bi-exponential decay and a coherent modulation, as illustrated in Figure 3(b). The PA signal amplitude was predominant. The signal amplitudes of the two satellite PB peaks were similar. The two decay times (*τ*_1_ and *τ*_2_) significantly differed at the ps and ns levels. The *τ*_1_ timescale shows a good agreement with the previously reported decay constant (∼2–3.5 ps) explained by the electron–longitudinal optical phonon scattering [19], [20]. After the fast decay (*τ*_1_), coherent modulation was observed, maintained for approximately 100 ps. The subsequent quasi-static state by the slow *τ*_2_ provided optical modulation without background from electron dynamics. Figure 3(c) depicts the QL number-dependent photocarrier dynamics. The faster relaxation time was similar for all samples. By contrast, the slower *τ*_2_ became slower as its thickness increased. Each decay time was obtained by fitting with the bi-exponential decay functions (Figure 3(d)). Note that the characteristics of the decay constants of PA and PB were almost the same. In other words, the observed changes in the NIR spectrum were caused by the excited electrons in conduction band and the subsequent line shape changes, which induced a second derivative-like TR signal.

**Figure 4:**
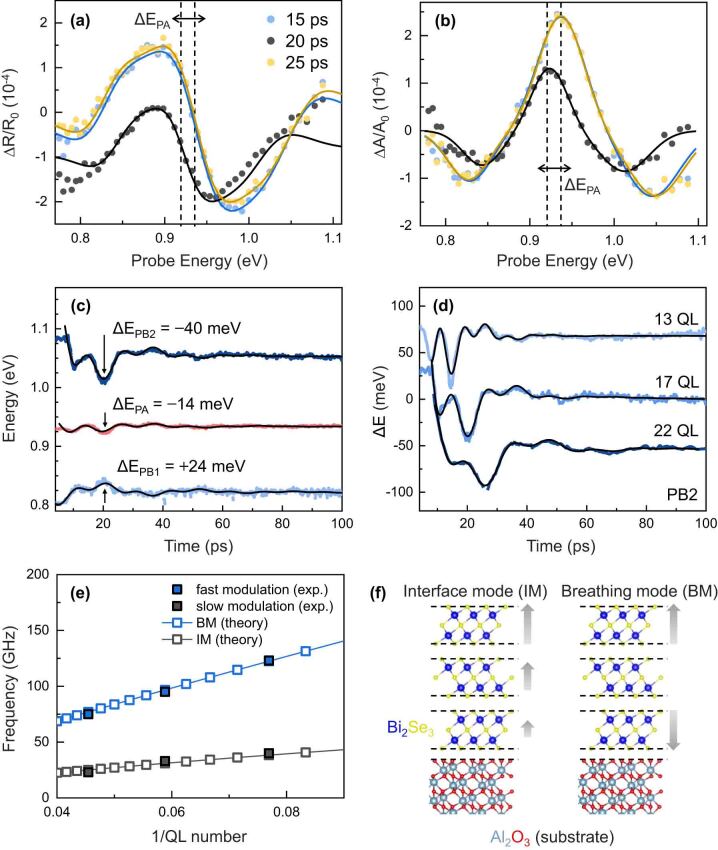
Ultrafast acousto-optic modulation: (a) representative TR spectra and (b) corresponding TA spectra at maximum and minimum modulations; (c) temporal traces of the resonant energy modulation for three resonant peaks; (d) time and number of QLs-dependent energy modulation for PB2 peak; (e) observed QL-dependent modulation frequency and simulation results based on the linear chain model with substrate effects; and (f) schematic of the layer vibrational modes measured through TR spectroscopy.

While the excited carriers persisted, a robust sinusoidal modulation with temporal coherence extending beyond 100 ps was observed. In Figure 4(a) and (b), representative transient reflectivity (TR) spectra and corresponding transient absorption (TA) spectra from the 17-QL sample at the selected time delay are depicted, highlighting the modulation’s maximum/minimum. Starting from 15 ps, reflectivity and absorption were significantly suppressed at 20 ps in the full probe range. The TR/TA spectra were subsequently recovered at 25 ps. The resonant energy was shifted (Δ*E*) through acoustic modulation. In Figure 4(b), the energy difference between the two satellite PB peaks (*E*_PB1_–*E*_PB2_) was reduced at 20 ps and recovered when compared to the spectra at 15 and 25 ps. For the second derivative TA, the *E*_PB1_–*E*_PB2_ reduction indicated the narrowing of the resonant optical transition by the ultrafast acoustic modulation. We visualized the ultrafast energy modulation of the interband transition by tracking the peak positions of the PB/PA in the TA spectra as shown in Figure 4(c).

The energy variation reached a maximum around 20 ps and was damped after three periods. In the 100 ps window in Figure 4(c), the TA spectra characteristics were quasi-static, that is, flat over the time window. To assess the energy difference caused by acoustic effects, we referenced the peak energies at 100 ps. The reductions in energy for the PB2 and PA bands were 40 and 14 meV, respectively, while the PB1 energy increased by 24 meV. Considering the second derivative-like TA spectra, the asymmetrical shift of the three peak energies can be interpreted by the shift and the broadening of the optical transition. The opposite sign of the energy variation for the PB1 and PB2 bands indicated the resonance narrowing, which implied the electronic band shrinkage. The asymmetric shifts between the PB1 and PB2 bands indicate energy gap change of the central resonant energy. This was also inferred from the fact that the energy variation asymmetry in the PB bands was similar to the shift value of the PA peak (i.e., Δ*E*_PB1_ + Δ*E*_PB2_ ∼ Δ*E*_PA_). Consequently, the ultrafast light-induced acoustic effects can shift and shrink the electronic band. Figure 4(d) illustrates the QL number-dependent ultrafast response of the resonant energy. The PB2 band was suitable for clearly comparing the number of QLs-dependent modulation speeds due to the largest amplitude of the energy modulation. Thicker samples showed more significant energy changes induced by vibration. The time-varying signal was well-fitted with two damped oscillations, and the fast and slow modulation components strongly depended on the QL number.

Figure 4(e) presents the frequencies obtained according to the QLs. The number of QLs dependent frequency in the layered system implied the unique interlayer vibrational modes caused by the weak vdW interactions explained by the linear chain model [21], [22], [23], [24], [25]. We calculated the frequency (*f*) as a function of the QL with the linear chain model having a substrate coupled case by reflecting the Bi_2_Se_3_ layered structure [26], [27]. We also used the force constants between QL–QL (*K*_0_) and QL-substrate (*K*_*S*_) as 6.34 × 10^19^ N m^−3^ and 1.27 × 10^19^ N m^−3^ [26]. We solved the linear chain model as follows:

(1) $$f_{n}=\pi^{-1}{\left(K_{0}/\mu \right)}^{1/2}\sin\left(q_{n}/2\right)$$

with constraint from the substrate as

(2) $$\tan\left(q_{n}/2\right)\tan\left(q_{n}N\right)=\frac{K_{s}/K_{0}}{2-K_{s}/K_{0}}$$

where, *µ* = 7.5 × 10^−6^ kg m^−2^ is the mass per unit area of each QL and *N* is the total number of QLs, respectively. The *q_n_* is defined by *q_n_* = *k_n_a* with *n*-th eigenmode wavevector (*k_n_*) and QL thickness (*a* = 1 nm). The finite *N* gives the discrete *q_n_*. The longitudinal sound velocity (*v*_*S*_) can also be obtained from the dispersion relation in Eq. (1) with the continuous medium limit (*ka* ≪ 1), which gives $f={\left(K_{0}/\mu \right)}^{1/2}ka/2\pi ={\left(K_{0}/\mu \right)}^{1/2}a/\lambda =v_{S}/\lambda$ . The obtained $v_{S}={\left(K_{0}/\mu \right)}^{1/2}a$ is 2.9 × 10^3^ m/s, which is in good agreement with previously reported values [28], [29], [30].

With given values of *v*_*S*_ and film thickness (*d = aN*), we can also utilize simple standing wave modes depending on the boundary condition to analyze the observed vibration frequencies. Employing the boundary condition of maximum or zero atomic layer displacement at the substrate interface, the frequency (*f*) can be determined by *f* = *n* *v*_*S*_/2*d* (free-standing, open-pipe mode) or *f* = (2*n*−1)*v*_*S*_/4*d* (rigid coupling with substrate, closed-pipe mode), where *m′* is an harmonic order. These two standing wave modes are also derived from Eq. (2) with boundary conditions of *K*_*S*_/*K*_0_ = 0 (open-pipe mode) or 2 (closed-pipe mode). In the case of *K*_*S*_/*K*_0_ = 0, the solution of atomic displacement function is *q*_*_n_*_ = *nπ*/*N*, from Eq. (2), which leads to $f_{n}\left(N\right)={\left(K_{0}/\mu \right)}^{1/2}\left(n/2N\right)=nv_{S}/2d$ . This describes the standing wave modes in relatively thick van der Waals films. This approximation can also be applied for another case of *K*_*S*_/*K*_0_ = 2, which leads to $f_{n}=\left(2n-1\right)v_{S}/4d$ . However, for instance, in a 13 QL (*d* = 13 nm) sample, the open-pipe (*f*_*n*_= *n* × 112 GHz) and closed-pipe (*f*_*n*_ = [2*n*−1] × 56 GHz) modes show significant differences from the observed fast (125 GHz) and slow (40 GHz) mode frequencies, respectively. Rather, the frequencies of the lowest mode of open-/closed-pipe mode are similar to that of the observed fast/slow mode, respectively, but the two modes cannot coexist. This discrepancy can be solved by using intermediate *K*_*S*_/*K*_0_ values (0.2 in this work). The *K*_*S*_/*K*_0_ value is less than 1, implying that the Bi_2_Se_3_ layers were loosely coupled with the substrate.

**Figure 5:**
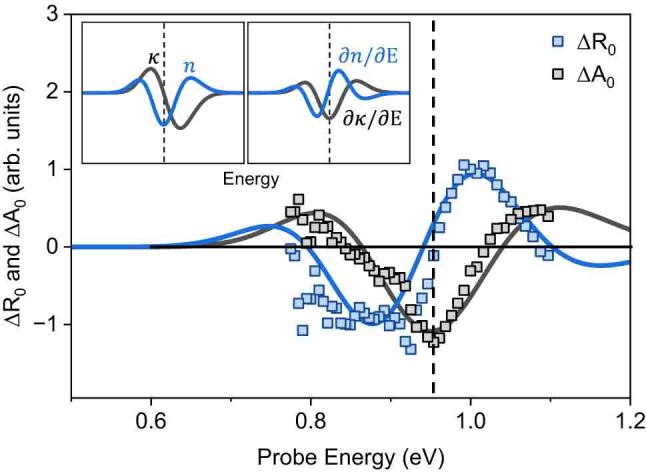
Probe energy-dependent amplitude of the interlayer vibration-induced reflectivity and absorption change, $\Delta R_{0}\left(E\right)$ and $\Delta A_{0}\left(E\right)$ with schematic *∂n* */* *∂E* and *∂κ* */* *∂E* guidelines. The inset schematic presents the changes of refractive index and derivatives dispersion at interband optical transition as indicated by vertical dashed lines.

Figure 4(e) displays the calculated eigenfrequencies of Eq. (1) with the boundary conditions of Eq. (2), demonstrating good agreement with the experimentally observed modulation frequencies. For the eigenmode *n*, the interface and breathing modes corresponded to *n* = 1 and 2, respectively. Figure 4(f) illustrates the interlayer vibrational motions for each mode. The ultrafast light-induced interlayer vibrations can alter the interlayer distance, resulting in a modulation of the energy and shape of the electronic band in Bi_2_Se_3_. The frequency of the detected oscillations in the photo-induced strain framework was independent of the probe energy, indicating confined acoustic waves in thin films. On the other side, the amplitude and phase of oscillation in TR signal depend on the probe energy due to variations in interband transitions, attributed to photoelastic coupling. In Figure 5, the amplitude of TR/TA by interlayer vibrations is presented as a function of probe energy. A resonance with a phase shift of *π* in two times is observed near the interband transition. By assuming that the TR and TA amplitude is predominantly influenced by variations in *n* and *κ* with respect to probe energy, respectively, the TR/TA are simply expressed using the Seraphin coefficients with atomic displacement (Δ*Q*), which is given by [31]

(3) $$\frac{\Delta R}{R_{0}}=\frac{\partial R}{\partial n}\frac{\partial n}{\partial E}\frac{\partial E}{\partial Q}\Delta Q\;\text{and}\;\frac{\Delta A}{A_{0}}=\frac{\partial R}{\partial \kappa }\frac{\partial \kappa }{\partial E}\frac{\partial E}{\partial Q}\Delta Q$$

As depicted in Figure 4, the phonon-induced transition energy shift and narrowing contribute to the modulation of energy-dependent refractive index (*n*) and extinction coefficient (*κ*), illustrated in the inset of Figure 5. Assuming that the optical transition is close to the Brillouin zone center (Γ point), we find that the dispersion of the deformation potential (*∂E*/*∂Q*) is negligible in comparison to the dispersion of changes in *n* and *κ* changes by Δ*Q* [31]. This assumption leads to a dependence of *∂n*/*∂E* and *∂κ*/*∂E* on the observed amplitude of the TR/TA modulation, as demonstrated in Figure 5. The experimental TR/TA amplitude by phonon is well reproduced with schematic *∂n*/*∂E* and *∂κ*/*∂E*. This observation strongly supports that the modulation of TR/TA was predominantly governed by changes in *n* and *κ*, respectively, driven by the interlayer vibration-induced transition energy shift and narrowing.

We further validate the observed interlayer vibration-induced changes in NIR resonance through DFT calculations. These calculations involved applying an out-of-plane strain to modify the interlayer distance. The DFT calculation were carried out for both relaxed and strained bulk Bi_2_Se_3_, considering spin–orbit coupling and vdW interactions. We used the Quantum Espresso [32], [33] package implemented in Material Square and the generalized gradient approximation with the Perdew–Burke–Ernzerhof functional [34], [35] to approximate the exchange calculations. To include spin–orbit interactions and vdW interactions [36] between the quintuple layers for all calculations, we chose a full-relativistic norm-conserving pseudopotential and applied the DFT-D3 method correction. The cut-off kinetic energy of the wave function was 85 Ry. The Brillouin zone was automatically sampled using 9 × 9 × 1 Monkhorst–Pack meshes. The convergence threshold and force were 10^−8^ eV and 10^−2^ eV/Å, respectively. After the convergence test, we performed variable-cell relaxation to obtain a fully relaxed bulk hexagonal unit cell and determine the atomic positions of Bi_2_Se_3_. The calculated lattice parameters for the fully relaxed bulk Bi_2_Se_3_ were *a* = *b* = 4.18 Å and *c* = 28.88 Å, consistent with previous reports [37], [38]. We also varied the interlayer distance between QLs and the lattice parameter *c* without changing the atomic position of each atom inside QL to model the atomic structure changes induced by light-induced strain. Subsequently, density of state (DOS) calculations were performed nonself-consistently with denser meshes of 27 × 27 × 3 using the tetrahedron method.

To compare with DFT calculations, we simulate the photo-induced strain amplitude by considering the electronic and thermal stresses generated by the deformation potential (*D*) and thermoelasticity [15]. We ignored the inverse-piezo effects that did not correspond to the Bi_2_Se_3_ crystal. Electronic stress was obtained with *D* and the excited carrier density (*n*_*ex*_) as −*D* *n*_*ex*_. We adopted herein a previously determined *D* of 22 eV [39]. Using an optical constant from Figure 1(b), *n*_*ex*_ was estimated as 5.8 × 10^19^ cm^−3^. For thermal stress (*σ*_*TE*_ = −3*βB*Δ*T*_*L*_) obtained by increasing the lattice temperature (Δ*T*_*L*_), we employed the linear expansion coefficient (*β*) and bulk modulus (*B*) from the literature [40], [41]. The Δ*T*_*L*_ changes were obtained from the simplified relation [42] of *F*_pump_/(*ξC*_*p*_), where *F*_pump_ is the pump fluence and *C*_*p*_ = 2.8 J K^−1^ cm^−3^ is the lattice heat capacity at 300 K [43]. The calculated electronic and thermal stresses were 0.11 and 0.03 GPa, respectively. The minus sign in the obtained stress indicated an expansion. The subsequent tensile strain along the longitudinal direction was 0.36 %. For the DFT calculation, we applied 1 % strain to emphasize the band structure changes under tensile strain.

**Figure 6:**
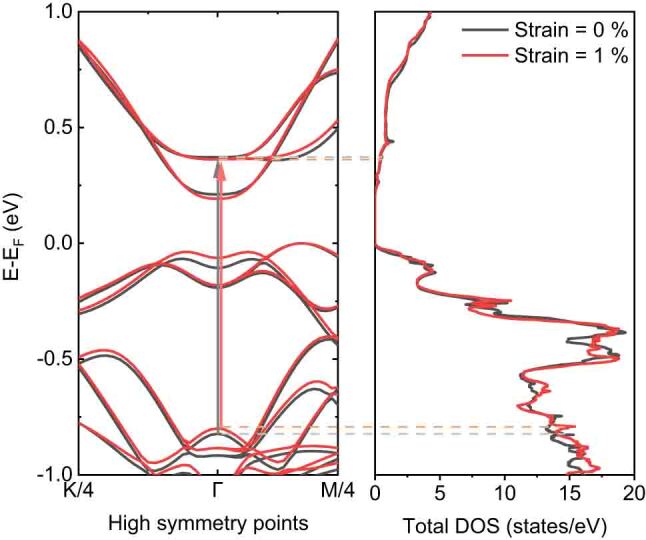
Electronic band structure and total density of states (DOS) of the relaxed (unstrained) and 1 % strained bulk Bi_2_Se_3_. The band structure was calculated along the path of the quarter of high-symmetry points *K*, Γ, and *M*. The interband transition energy of the relaxed Bi_2_Se_3_ was determined as 1.19 eV, which decreased to approximately 30 meV when 1 % of tensile strain was applied. This result supported the pump–probe experimental result well.

In Figure 6, we display the calculated electronic band structure and the total density of states (DOS) for both relaxed and 1 % strained Bi_2_Se_3_. The band structure near the Γ point contracts when subjected to a 1 % tensile strain, resulting in a reduction of the energy gap by approximately 30 meV. Comparing this to the observed energy shift of about 14 meV, the theoretical calculations align well with the experimental results. By plotting the band structure and the DOS together, we found optical transition of approximately 1 eV for the unstrained Bi_2_Se_3_, which was consistent with the results of previous studies [16], [18]. This value slightly decreased due to the band shift. The narrowing of the NIR resonance through interlayer vibration was attributed to the band shrinkage near the Γ point under a longitudinal strain. The electronic band shrinkage by the interlayer vibrations was caused by the observed AO effects. In other words, the SOC effect changed when the Coulomb interaction between Se atoms changed [25], [41], [44]. Throughout this process, electronic bands may contract in momentum space while maintaining the total DOS, which attributes to the observed ultrafast modulation of NIR spectra in Bi_2_Se_3_ vdW thin films by light-driven interlayer vibrations along longitudinal direction.

## 3 Conclusions

In this study, we utilized ultrafast NIR spectroscopy and DFT calculations to showcase acousto-optic effects at the broad NIR range in Bi_2_Se_3_ vdW thin films. Ultrafast lasers were employed to induce interlayer vibrations in the range of hundreds of GHz. Thickness control provided frequency tunability from sub-GHz to sub-THz within the crystal. These ultrafast acoustic interactions persisted for 100 ps, a duration sufficiently long compared to the lattice vibration period. The vibrational modes demonstrated high speed, rendering them suitable for narrow bandwidth applications. Importantly, the incoherent electronic background was relaxed under ultrafast (∼2 ps) and ultraslow (∼ns) conditions, offering an almost flat incoherent offset during the acoustic modulation. The excitation of the coherent acoustic phonons was generally based on the excited charge interactions; hence, a flat electronic background was difficult to achieve in the all-optical modulation scheme [12], [13], [45], [46], [47], [48], [49].

Our results demonstrate the interaction of the photoexcited carriers with the lattice, launching unique interlayer vibrations coupled with resonant optical transitions near 1.0 eV. In the microscopic view, the changes in the interlayer distance altered the gap between the electronic bands, showing a corresponding NIR resonance. This led to the resonance energy shift. The NIR resonance indicated asymmetric shift that manifested the line shape modulations. This scheme enables for all-optical modulators by generation and detection of coherent interlayer vibrations, highlighting the potential for future modulators with high frequency and integrability at the nanophotonic scale. Particularly in the NIR spectral range, where Bi_2_Se_3_ demonstrates high photo-responsivity [50], [51], this approach holds promise for superior performance in signal detection and processing. However, the modulation depth in reflectivity/absorption induced by interlayer vibrations is on the order of 10^−4^ in the NIR spectrum, which is small compared to the commercial AO devices. Hence, achieving a large amplitude for such application is essential, reaching a level that is compatible with other conventional measurement systems. The acousto-optic effects can be enhanced by applying bias to promote phonon generation through drifting electrons [52], [53], [54], optomechanical cavities [55], and emerging integrated platforms [56], as the observed interactions between interlayer vibrations and photons occur within a few tens of nanometers.
